# Novel targeted therapies and immunotherapy for advanced thyroid cancers

**DOI:** 10.1186/s12943-018-0786-0

**Published:** 2018-02-19

**Authors:** George E. Naoum, Michael Morkos, Brian Kim, Waleed Arafat

**Affiliations:** 1 Department of Radiation Oncology, Harvard Medical School, Massachusetts General Hospital, 55 Fruit St, Boston, MA 02114 USA; 2 Alexandria Comprehensive Cancer center, Alexandria, Egypt; 3 Department of Endocrinology, Rush University, 1900 W Polk St, Room 801, Chicago, IL USA; 4 Department of Endocrinology, Thyroid Cancer Program, Rush University, Jelke Building, Room 604, 1735 W Harrison St, Chicago, IL 60612 UK; 5 University Of Alexandria, Clinical oncology department, Alexandria, Egypt; 6 Department of Radiation Oncology, University of Alabama at Birmingham, 1720 2nd Ave S, Birmingham, AL 35294 UK

## Abstract

Thyroid cancer is a frequently encountered endocrine malignancy. Despite the favorable prognosis of this disease, 15–20% of differentiated thyroid cancer (DTC) cases and most anaplastic types, remain resistant to standard treatment options, including radioactive iodine (RAI). In addition, around 30% of medullary thyroid cancer (MTC) cases show resistance after surgery. The evolving understanding of disease-specific molecular therapeutic targets has led to the approval of two targeted therapies (Sorafenib and Lenvatinib) for RAI refractory DTC and another two drugs (Vandetanib and Cabozantinib) for MTC. These advanced therapies exert their effects by blocking the MAPK pathway, which has been widely correlated to different types of thyroid cancers. While these drugs remain reserved for thyroid cancer patients who failed all treatment options, their ability to improve patients’ overall survival remain hindered by their low efficacy and other molecular factors. Among these factors is the tumor’s ability to activate parallel proliferative signaling pathways other than the cascades blocked by these drugs, along with overexpression of some tyrosine kinase receptors (TKR). These facts urge the search for novel different treatment strategies for advanced thyroid cases beyond these drugs. Furthermore, the growing knowledge of the dynamic immune system interaction with tumor microenvironment has revolutionized the cancer immune therapy field. In this review, we aim to discuss the molecular escape mechanisms of thyroid tumors from these drugs. We also highlight novel therapeutic options targeting other pathways than MAPK, including PI3K pathway, ALK translocations and HER2/3 receptors and their clinical impact. We also aim to discuss the usage of targeted therapy in restoring thyroid tumor sensitivity to RAI, and finally turn to extensively discuss the role of immunotherapy as a potential alternative treatment option for advanced thyroid diseases.

**Electronic supplementary material:**

The online version of this article (10.1186/s12943-018-0786-0) contains supplementary material, which is available to authorized users.

## Background

For the past several decades thyroid cancer has been the most common endocrine tumor, with a ~ 5% increase in incidence each year in the USA [1, 2]. The vast majority of thyroid cancers arise from thyroid follicular cells (93%) and are well-differentiated (DTC). Most of these are categorized on histologic grounds as being papillary thyroid cancers (PTC), or less commonly as follicular thyroid cancers (FTC), the latter being associated with a worse prognosis. Poorly differentiated forms with even more aggressive clinical behavior are relatively uncommon and the highly fatal anaplastic thyroid cancers (ATC) are fortunately rare [3, 4]. Parafollicular cell-derived medullary thyroid cancers (MTC) are also rare, comprising ~ 3% of thyroid carcinomas [5].

The standard therapeutic approach to all thyroid cancers includes surgery, with radioactive iodine (RAI) being offered to some patients with follicular cell-derived thyroid cancers [6–8].

A small fraction (< 10%) of DTC as well as many MTCs and almost all ATCs are not cured by standard therapy, instead spreading to distant metastatic sites. If grouped together as “advanced thyroid cancers,” patients with these aggressive forms have a less than 50% 5 year survival rate in contrast to the ~ 98% 5-year survival for iodine-sensitive DTC patients [9].

Recently, a number of scientific advances have illuminated some of the molecular pathways responsible for thyroid cancer. This growing knowledge raises the hope that it will soon be possible to develop specific therapeutics tailored to these molecular changes [10]. While multiple kinase inhibitor drugs (MKIs) targeting MAPK pathway have had some clinical benefit, improvements in overall survival is still debatable [11]. Both the presence of tumoral intrinsic resistance mechanisms to these MKIs, as well as the systemic toxicity of the drugs have limited their clinical benefits [12]. Therefore, novel approaches must be explored for advanced thyroid cancers.

This review article considers the major therapeutic strategies currently being investigated in the field of advanced thyroid cancer, focusing on approaches with not only pre-clinical but also clinical trial data. We aim to discuss novel and experimental MKIs for advanced thyroid cancers, radioactive iodine (RAI) resensitization and finally a section on immunotherapy.

It is to be noted that search strategy and selection criteria and references for this Review were identified through searches of PubMed, “clinicaltrials.gov”, and oncology conferences’ websites with the search terms “thyroid cancer”, “targeted therapy”, “MAPK”, “radioactive iodine refractory thyroid cancer”, and “immunotherapy for thyroid cancer” since inception. Only papers published in English were reviewed. The references were included based on their pertinence to the scope of this Review

## MKIs in advanced thyroid cancer

**Fig. 1:**
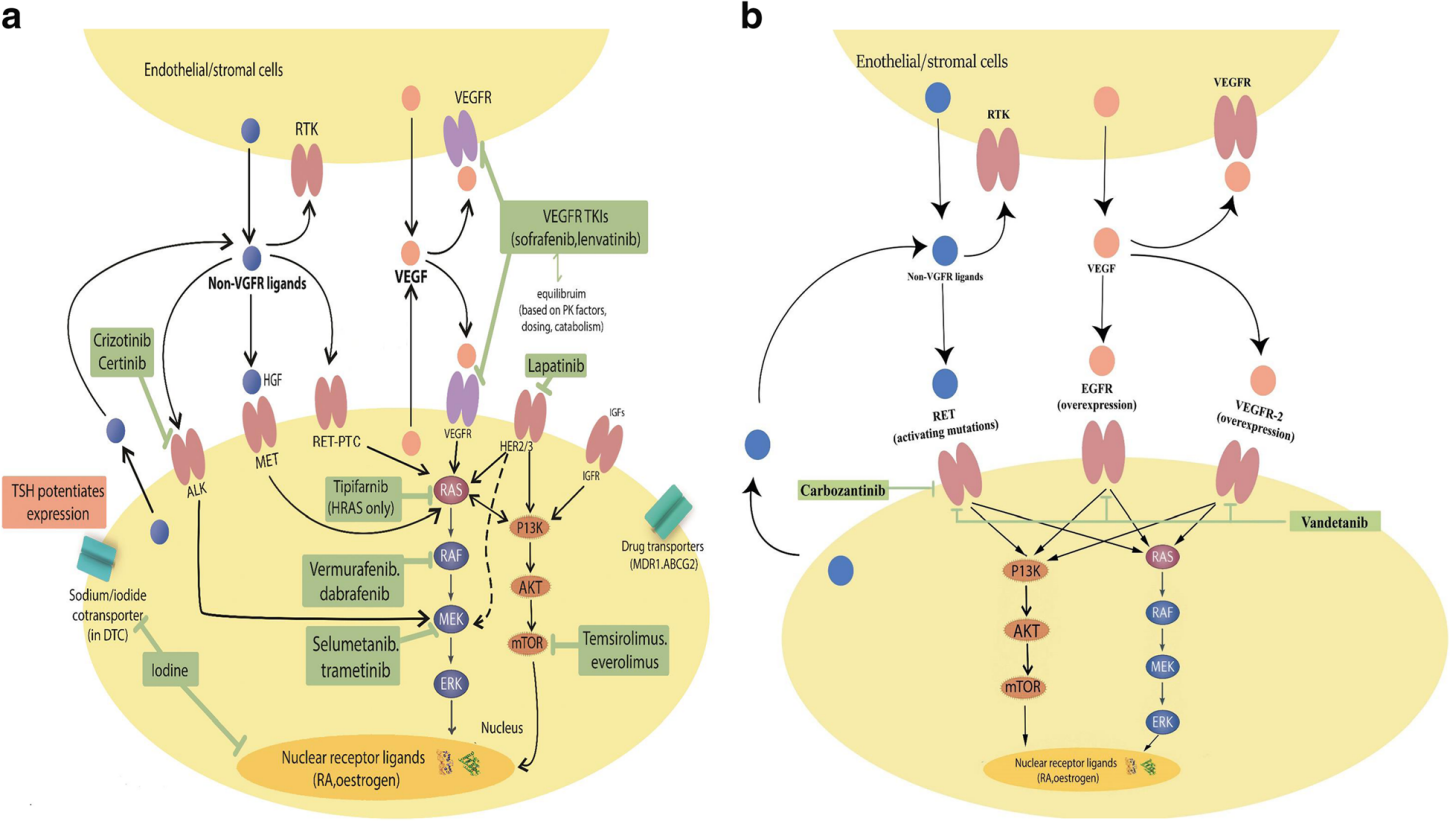
Dominant signaling pathways involved in thyroid cancers, and clinically relevant inhibitors: **a**: MAPK and PI3k tyrosine kinase Receptors are shown in DTC cells, along with their respective ligands and downstream cascades. All clinically approved drugs are highlighted in green. The cross talk between MAPK and PI3k is shown through RAS and represent a tumoral escape mechanism from known multiple kinases inhibitors acting on B-RAF. ALK and Her2/3 receptors are shown with their downstream signaling pathways representing another tumoral escape mechanism from conventional drugs working on RAS and RAF. Note that stromal and endothelial cells, as well as cancer cells, participate in VEGFR and other signalling pathways that contribute cancer proliferation. **b** the regulating pathways of MTC cells are shown with the same cross talk between MAPK and RAS. MTC approved targeted therapy with their corresponding targets and receptors

The MAPK signaling pathway (Fig. 1) is one of the most extensively studied pathways in oncology [13]. Upon pathologic activation of different tyrosine kinase receptors (TKR), a cascade of downstream events in this pathway ultimately leads to cell proliferation, differentiation, and survival. Data from the cancer genome atlas (TCGA), has allowed better classification and molecular characterization of PTC, using integrated multiplatform data with a large sample size [14]. According to this data, PTC has been classified as an MAPK driven tumor with the two major signaling drivers being *BRAF*^V600E^ and mutated *RAS. In addition, mutations in PI3K pathways, as well as a few genetic somatic mutations and fusion alterations have been reported (with lower frequency) in PTC* [14]*. In* Table 1*, major drivers of thyroid cancer are identified.*

**Table 1:** Mutations in thyroid cancers

Thyroid cancer type	Mutation	Description and significance	Reference
Differentiated thyroid cancers	*B-RAF*	*B-RAF* point mutations (particularly glutamate substitution for valine at residue 600, V600E) are present in 30–70% of patients with PTC	[96–104]
	RAS	RAS oncogene mutations are manifested in 15–20% of PTC and 40–50% of FTC	[105–111]
	RET/PTC rearrangements	*RET/PTC1* and *RET/PTC3 together* comprise > 90% of RET/PTC mutations in thyroid cancer); *RET/PTC2* represents less than 5%. Combined, RET/PTC rearrangements are found in ~ 13% of PTC	[112–119]
	PAX8-PPARγ translocations	PAX8 drives the expression of many thyroid-specific genes such as those encoding thyroglobulin, the sodium iodide symporter and thyroid peroxidase. The PAX8-PPARγ translocations are found in 35% of FTC and vascular invasion and tumor proliferation have been linked to these translocations.	[120–125]
	Telomerase reverse transcriptase gene (*TERT*) mutations	Found to be overexpressed in DTC; 11% of FTC and 16–40% of PTC (frequently in association to *B-RAF* mutations) were found to bear *TERT* mutations. Coexistence of TERT with BRAF mutation was concluded to have the worst prognosis for DTC patients especially those with PTC subtype [126]. However, studies examining TERT as a therapeutic target are lacking.	[126–128]
	PIK3CA	PIk3CA gene encodes for catalytic subunits in PI3K leading to activating the proliferative cascade of PI3K/AKT pathway. Increased copy numbers of this gene has been found in 24% of FTC and 42% of ATC.	[25]
	PTEN	PTEN normally antagonizes and terminates the signaling of the PI3K/Akt pathway. It was found that around 12% of ATC exhibit mutated or deleted PTEN genes and hence over activation of the proliferative PI3k pathway and more tumoral aggressiveness.	[25]
Medullary thyroid cancers	RET mutations	> 60% of MTC have been linked to somatic RET oncogene mutations.	[129]

At the time of this writing, the FDA has approved four different drugs targeting the Mitogen-Activated Protein Kinase (MAPK) signaling pathway in the treatment of advanced thyroid cancers [15, 16]. These include Lenvatinib and Sorafenib for advanced, recurrent, and RAI-refractory (RAI-R) DTC; and Cabozantinib and Vandetanib for MTC. Also several MKIs targeting MAPK are being studied in advanced thyroid cancers but none of them have been FDA approved yet (Table 2). The approved targeted therapeutics exert their anti-tumoral activity mainly by competing with ATP at its binding site on the tyrosine kinase receptor (TKR) and partially by blocking several central mediators of MAPK pathway (Fig. 1). The mechanisms of these MKIs and their clinical impact have been extensively reviewed [11, 17–19]. As the developmental history and clinical data for the FDA-approved MKIs have been recently reviewed elsewhere [11], that aspect is not considered here.

**Table 2:** Other MKIs for MAPK are being tested for different types of thyroid cancer but at the time of writing this article, none have reached phase III trials

Drug	Drug targets	Phase	Dosage	Patients	Partial response RR (%)	Progression-free survival PFS (months)	Adverse effects (%)	Drug discontinuation
Multikinase inhibitors								
Axitinib [130]	VEGFR, PDGFR, c-kit	II	starting dose of 5 mg twice daily	60	30	18.1	Fatigue (50%), diarrhea (48%), nausea (33%), anorexia (30%), hypertension (28%), stomatitis (25%), weight loss (25%), and headache (22%)	32 patients, 8 of them due to treatment side effects
Motesanib [131]	VEGFR, PDGFR, c-kit	II	125 mg/day orally for up to 48 weeks	93	14	9.3	Diarrhea (59%), hypertension (56%), fatigue (46%), and weight loss (40%)	61 patients, 12 of them due to treatment side effects
Sunitinib [132]	PDGFR, FLT3, c-kit, VEGFR, RET	II	37.5 mg/day orally	35	31	12.8	Neutropenia (34%), fatigue (11%), HFS (17%), diarrhea (17%), and leukopenia (31%)	4 patients due to treatment side effects
Pazopanib [133]	VEGFR, PDGFR, c-kit	II	800 mg/day orally in 4-week cycle	37	49	11.7	Fatigue (78%), skin and hair hypopigmentation (75%), diarrhea (73%), and nausea (73%)	27 patients, 2 of them due to treatment side effects
Dovitinib [134]	FGFR, and (VEGFR)	II	500 mg/day orally for five consecutive days, followed by a 2-day rest every week.	40	20.5	5.4	Diarrhea (54%), anorexia (36%), vomiting (26%), fatigue (23%), and nausea (21%)	12 patients
Imatinib [135]	BCR-ABL, PDGFR-α, PDGFR-β, c-fms, c-Kit, and RET	II	600 mg/day orally	15	0	NR	Hypothyroidism (60%), rash, malaise, and laryngeal mucosal swelling (13%)	10 patients, 3 of them due to treatment side effects
Selumetinib (AZD6244) [43]	MEK-1/2 (one of MAPK), RAS, V600E BRAF	II	100 mg twice daily for 28-days cycles	39	3	8	Rash (77%), fatigue (49%), diarrhea (49%), and peripheral edema (36%)	Only 6 patients due to treatment side effects
Selective BRAF inhibitors								
Dabrafenib [136]	BRAF	I	150 mg twice daily or 100 mg three times daily	14	29	11.3	skin papillomas (57%), hyperkeratosis (36%), alopecia (29%), elevated lipase (*7%*), grade 3 elevated amylase (*7%*), grade 3 fatigue (*7%*), grade 3 febrile neutropenia (*7%*), and grade 3 cutaneous squamous cell carcinoma (*7%*)	None
Vemurafenib [137]	BRAF	II	960 mg orally twice daily	51	35	15.6	squamous cell carcinoma of the skin (23.5%), lymphopenia (8%), and increased γ-glutamyl-transferase (8%)	

## MAPK and PI3K pathways cross talk and tumor escape mechanism

To date, efforts based on pharmaceutically blocking MAPK increased progression-free survival in DTC and MTC [20]. A recent single study showed that Lenvatinib achieved improvement in overall survival in older patients (> 65 years old) with advanced DTC in comparison to younger patients [21]. Also the same study showed that this old age group suffered from higher toxicity in comparison to younger patients, rendering debatable clinical decisions in today’s practice. Other than systemic toxicity, the development of tumoral escape mechanisms to these drugs represents an additional limitation that should be considered more closely. Known tumoral escape cascades and mechanisms from MKI drugs include induction of either alternative signaling pathways or to tyrosine kinase receptor (TKR) upregulation on the tumoral cell surface [22–24]. As depicted in Fig. 1, the MAPK and PI3K-AKT cascades are overlapping, with upregulation of either pathway leading to the same end-result of tumor cell survival and proliferation. Some of the major PTC drivers, the RAS and RET/PTC oncogenes are essential components of MAPK pathway, with cross talk effects with the PI3K-AKT cascade (Fig. 1). The role of PI3K-AKT cascade in initiating and promoting the progression of thyroid tumors has been reviewed by Saji et al. [25, 26]. Activation of AKT leads to downstream activation of different proliferative targets including: forkhead family of transcription factor (FoxO), mammalian target of rapamycin (mTOR) and others [26].

Recent data, including the thyroid cancer gene atlas, suggests that other novel escape mechanisms may also exist, e.g. TKRs in other pathways have been found to be expressed in thyroid tumor cells, with evidence of downstream pro-proliferative actions [14, 27–29]. For example, studies on patient-derived thyroid tumors from patients with advanced cancer have demonstrated that a substantial percentage exhibit the increased presence of HER2/3 receptors as well as ALK rearrangements. These mutations tend to serve as tumoral escape mechanisms from the currently used MKIs through activating MEK the downstream effector of RAF [27] as depicted in Fig. 1. HER2/3 has been found to be capable of activating PI3k cascade as well (Fig. 1). In the light of these facts, a number of strategies using multiple pathways targeting agents are under study.

### Upstream targeting of tumoral escape cascade

#### 1. Targeting (ERBB-HER2/3)

A novel line of investigation involves targeting proposed upstream elements relative to MAPK/PI3K. HER2 (ErbB2) and HER3 (ErbB3) are tyrosine kinase receptors and member of the Epidermal growth factor receptor (EGFR) family [27]. Their activation leads to downstream activation of both MAPK and PI3K cascade (Fig. 1). Preclinical data has shown that targeting these receptors by MKIs has represented an effective strategy, especially for breast cancer where these receptors are abundant. An escape mechanism identified in BRAF-mutant cells treated by Vemurafenib, is an overexpression of HER2/3 receptors and subsequent activation of mTOR cascades and MAPK [30] (Fig. 1). Therefore, current clinical efforts are assessing the role of HER receptors targeting in advanced thyroid cancer. NCT01947023 is a phase I trial assessing Lapatinib (HER2/3 blocker) in combination with Dabrafenib (BRAF inhibitor) for patients with advanced DTC and its primary results were published in ASCO 2017. Investigators included 15 evaluable patients with BRAF V600 mutations, 13 patients had DTC and 2 had ATC, prior MKI therapy was administered in 9/15 patients. Dabrafenib was given to all patients as 150 mg bid starting 2 weeks prior to Lapatinib. Doses of daily Lapatinib were escalated in a standard 3 + 3 design at (1) 750 mg; (2) 1250 mg; (3) 1500 mg. The reported partial response rate is 60% with a median PFS of 15 months (range, 2–34+ months). Only 1/15 patients developed grade III treatment-related toxicity, while dose-limiting toxicities were reported in only 1 patient with ATC and were unlikely related to drugs [31]. In another trial, the pan-ERBB Inhibitor Neratinib (NCT 03065387) is being assessed for its effect in advanced solid tumors harboring any HER mutation, including thyroid cancer.

#### Targeting ALK translocations

Recently, ALK gene translocations were identified in patient-derived thyroid cancer cells [14] . In contrast to *different ALK* translocation fusions, identified in other tumor types, the *STRN-ALK* fusions encoding for striatin were identified in thyroid cancer cells [32]. These hybrid mutations are found in up to 4% of ATC and 9% of poor differentiated thyroid cancer [33]and lead to continuous activation of proliferating MAPK pathway through MEK activation [34] (Fig. 1). Two clinical reports describe the effect of selective ALK inhibitors in the setting of ATC. Godbert et al. reported in a case report the effect of Crizotinib (an ALK inhibitor that is FDA-approved for ALK positive non-small cell lung cancer) in a 71 year-old female with ALK-rearrangement ATC [35]. This patient with stage IV, T4/N1A disease failed the initial treatment of concomitant chemoradiotherapy postoperatively (Cisplatin, Etoposide, and radiotherapy) with progression of metastatic pulmonary nodules. The second line chemotherapy of carboplatin and etoposide was administered with no clinical benefit after 3 cycles. Strikingly, as a last step approach, genetic profiling revealed ALK overexpression with intact EML4 gene. Based on this finding, Crizotinib (ALK inhibitor) was started and 3 months post-treatment, CT showed 90% regression in pulmonary nodules (RECIST criteria) and exceptional ECOG (Eastern Cooperative Oncology Group) performance status [35]. NCT02289144 is the only recruiting clinical trial, so far, aiming to assess the role of Ceritinib in patients with advanced ATC with positive ALK abnormalities.

### Downstream targeting of tumoral escape cascade

#### Targeting mTOR

The growing knowledge of involvement of PI3K pathway in thyroid tumor cells pathogenesis and escape from conventional targeted therapy led to closer studying several components of this pathway. Among these components is mTOR (mammalian target of rapamycin), which when inhibited has led to cell proliferation suppression [36].

Two mTOR inhibitors have sufficiently favorable trial data that they have been approved by the FDA for clinical usage for other cancers: Temsirolimus in advanced renal carcinoma and Everolimus for advanced renal carcinoma, metastatic breast cancer, and progressive endocrine tumors of pancreatic origin (PNET) [36]. Given observed clinical benefits of these drugs, and the belief that mTOR activation is a therapeutic target in thyroid cancer [37], these agents are being tested in the setting of advanced thyroid cancer. Early results from Phase II thyroid cancer trials give reason for hope. For example, NCT01025453, a Phase II study assessing the effect of Temsirolimus with Sorafenib in RAI-R thyroid cancer patients [38]. Out of 37 patients, eight patients achieved partial response (PR), 21 had stable disease, while one patient developed progression and the remaining seven were un-evaluable. These results were independent from RAS or RAF mutation and reflect the importance of considering mTOR targeting as a promising approach for RAI-R and advanced thyroid cancer patients. The estimated completion date of this trial is December 2017 for final data collection and primary outcome measurement. In addition, NCT01141309, a Phase II Study evaluating the combination of Everolimus (mTOR inhibitor) and Sorafenib was presented at ASCO 2015 [39]. Out of 41 patients with different types of progressive thyroid cancers included, only 38 were evaluable for the primary endpoint of clinical benefit at the date of publishing the results. In this cohort, 21 patients (55%) had PR, 14 patients (37%) achieved stable disease (SD), and three patients (8%) had progressive disease (PD). The subgroup and general toxicity assessment of this combination showed better response and less toxicity when compared to Sorafenib monotherapy, especially in the DTC subgroup.

NCT01263951 is an ongoing trial (enrolling at the time of this writing) testing the effect of this combination in DTC patients who progressed on Sorafenib therapy alone. Its preliminary results presented at ASCO 2015 showed remarkable reduction in Sorafenib-induced hand-foot syndrome (HFS) as a side effect, with SD as the best response achieved in most patients [40].

NCT02143726, an enrolling phase II study, assesses the efficacy and safety of Sorafenib with or without Everolimus in treating patients with advanced, RAI-R Hurthle cell thyroid cancer.

Finally, the abundance of PI3K/Akt/mTOR abnormalities in ATC spots this pathway as an attractive target for group of patients suffering from ATC [25]. NCT02289144 is a recruiting trial aiming to assess the effect Ribociclib (targeting Retinoblastoma (RB) mutation) and Everolimus (mTOR inhibitor) in metastatic Rb + ATC patients. The rationale of targeting Rb in those patients is the abundance of intact RB in ATC patients [41]. However, the awaited results of this trial may suggest new treatment strategies for ATC patients since retinoblastoma gene is still not considered a therapeutic target in ATC patients.

#### MEK targeting

As mentioned earlier, ALK translocation and HER2/3 mutations are capable to induce their pro tumor proliferative effect through MEK, a downstream effector of MAPK pathway (Fig. 1) [42]. This fact has attracted several trials to assess the effect of MEK blockage regardless of underlying genetic or molecular mutation.

The results of the phase II trial of Selumetinib (MEK 1–2 inhibitor) [43] yielded a tolerable safety profile in RAI-R PTC patients. However, it is to be noted that the effect of Selumetinib on progression-free survival (PFS) was affected by BRAF mutation status; where BRAF V600E mutants had a longer median PFS compared with patients with BRAF wild-type (WT) tumors. This suggest a preliminary evidence of stratified beneficence of Selumetinib based on underlying genetic abnormalities. Therefore more clinical trials with larger patients cohorts are addressing the efficacy of this drug in order to provide more clear evidences. NCT01843062 is currently an ongoing phase III trial aiming to assess the efficacy of Selumetinib vs placebo in DTC patients.

Other MEK inhibitors are being also assessed in advanced thyroid cancers. The primary results of NCT02034110 assessing Efficacy of Dabrafenib (BRAF inhibitor) and Trametinib (MEK inhibitor) in 16 patients with BRAF V600E (ATC) were published in ASCO 2017. The overall response rate (ORR) was reported to be 69% (11/16 patients). This regimen showed a controversial safety profile with grade 3–4 toxicities occurring in ≤19% of patients. However all of these toxicities were manageable [44].

The controversial preliminary results of NCT01723202 showed that adding Trametinib to Dabrafenib did not result in statistical significance in PR, ORR, SD, PFS as compared to Dabrafenib alone in BRAF mutated PTC patients [45]. These contradicted results open the question if the concept of MEK inhibitionalone or the underlying tumoral phenotype is the reason behind these results. However, the final results of these trials and comparative analysis of MEK blocking effect in different thyroid tumor types will add more solid evidence.

## Iodine Resensitization using different strategies including MKIs

Radioactive iodine (RAI) is widely used in the treatment of advanced follicular cell-derived thyroid cancers, but downregulation of the sodium-iodide symporter gene (*SLC5A5*, more commonly known as NIS) leads to resistance or RAI-R disease [46]. A novel therapeutic strategy has been to pharmacologically induce re-expression of NIS, i.e. “redifferentiating” the cells such that they can be treated with radioiodine.

In this regard, in vitro studies showed that Retinoids (Vitamin A-derived retinoic acids) are able to induce thyroid tumors redifferentiating effects like induction of 5′-deiodinase [47], increased expression of NIS mRNA [48], and increased thyroglobulin level (Tg), known to be lost in de-differentiated thyroid cancers [47]. Several clinical studies followed that principle for inducing RAI resensitization [37, 49–52]. The results of these studies indeed showed that retinoic acid therapy could, to a certain degree, induce RAI reuptake. Despite that finding, a true clinical beneficence in controlling underlying thyroid tumors has not been widely demonstrated, rendering the clinical application of retinoids in daily practice a doomed option.

A related line of investigation has examined whether Pax-8/PPARγ agonists might, in theory, upregulate NIS in RAI-R tumors. Based on pre-clinical data positively correlating Pax-8 (Paired box-8) and NIS promotor activity [53], along with the observation that PAX8-PPARγ translocations are present in 35% of FTC. Accordingly, a phase II study demonstrated that the Pax-8-PPARγ agonist Rosiglitazone (4 mg daily for 1 week, then 8 mg daily for 7 weeks) was associated with improved I-131 uptake in 4 out of 10 subjects [54].

Recently, the observation that constitutive activation of MAPK signaling causes transcriptional inhibition of thyroid hormone biosynthesis genes including NIS has led to the investigation of certain selective MAPK blocking agents as RAI resensitizer agents. [55].

In a phase II ‘proof of concept’ trial, Selumetinib (a MEK inhibitor) increased iodine uptake in 12 of 20 patients (quantitative I^124^ uptake). Eight of the 12 patients reached the dosimetry threshold for RAI therapy [56]. The genetic subgroup analysis showed that all patients with RAS-mutated DTC (5 patients) responded to Selumetinib, while only four out of nine patients with BRAF mutation had a response. This difference could be attributed to either the effect of BRAF mutation on normal thyroid gene expression or to BRAF-induced TGFB (Transforming Growth Factor B) signaling, which represses NIS expression [57]. Two ongoing trials, NCT01843062 and NCT02393690, both placebo-controlled trials with Selumetinib in locally-advanced, recurrent, or metastatic thyroid cancer will add more data to this existing pool of evidence, as well as a similar trial with the MEK inhibitor Trametinib, (NCT02152995).

Dabrafenib (BRAF inhibitor) restored RAI uptake in PTC advanced patients [58]. Out of 10 selected patients, 60% (*n* = 6) showed increase RAI-131 uptake with 2/6 having partial response.

## Immunotherapy in thyroid cancer

Several preclinical studies suggested promise for immunotherapy in the treatment of advanced follicular cell derived cancer as well as for MTC. While this general approach has been less developed in terms of clinical trial data than the MKI studies, there are several ongoing immunotherapy trials with clinical promise.

### Changes in immune system relevant to thyroid cancer

**Fig. 2:**
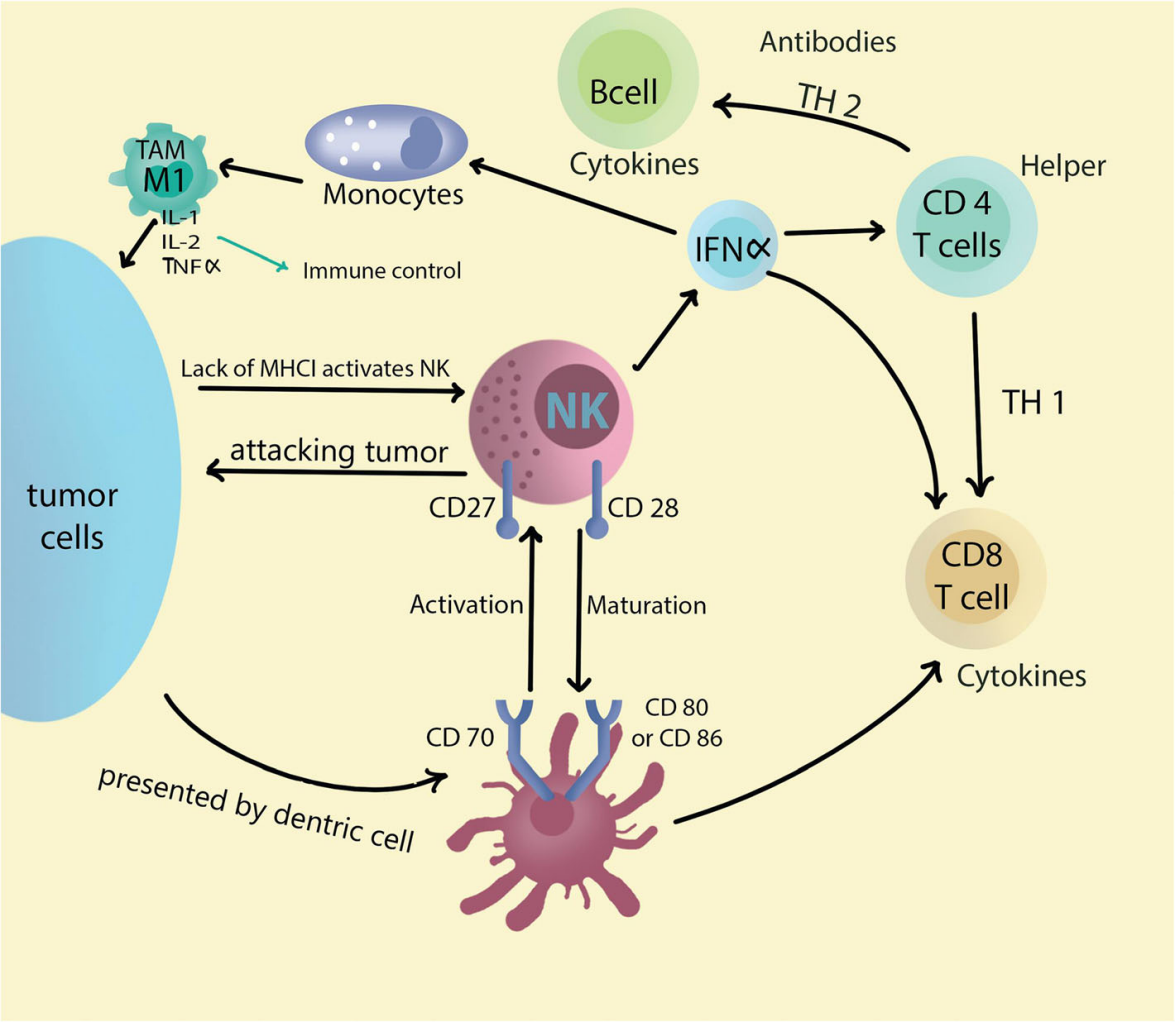
Tumor immune surveillance and host early response to tumor microenvironment: The lack of MHC I on tumor cells activates Natural killer cells. Dendritic cells present tumor antigens to cytotoxic T cells which exhibit a cytotoxic activity on dividing malignant cells. Note in tumor surveillance phase that tumor associated macrophages (TAM) present in tumor microenvironment are of anti-tumoral M1 phenotype and also the expressed cytokines in the medium are of immune stimulatory type

**Fig. 3:**
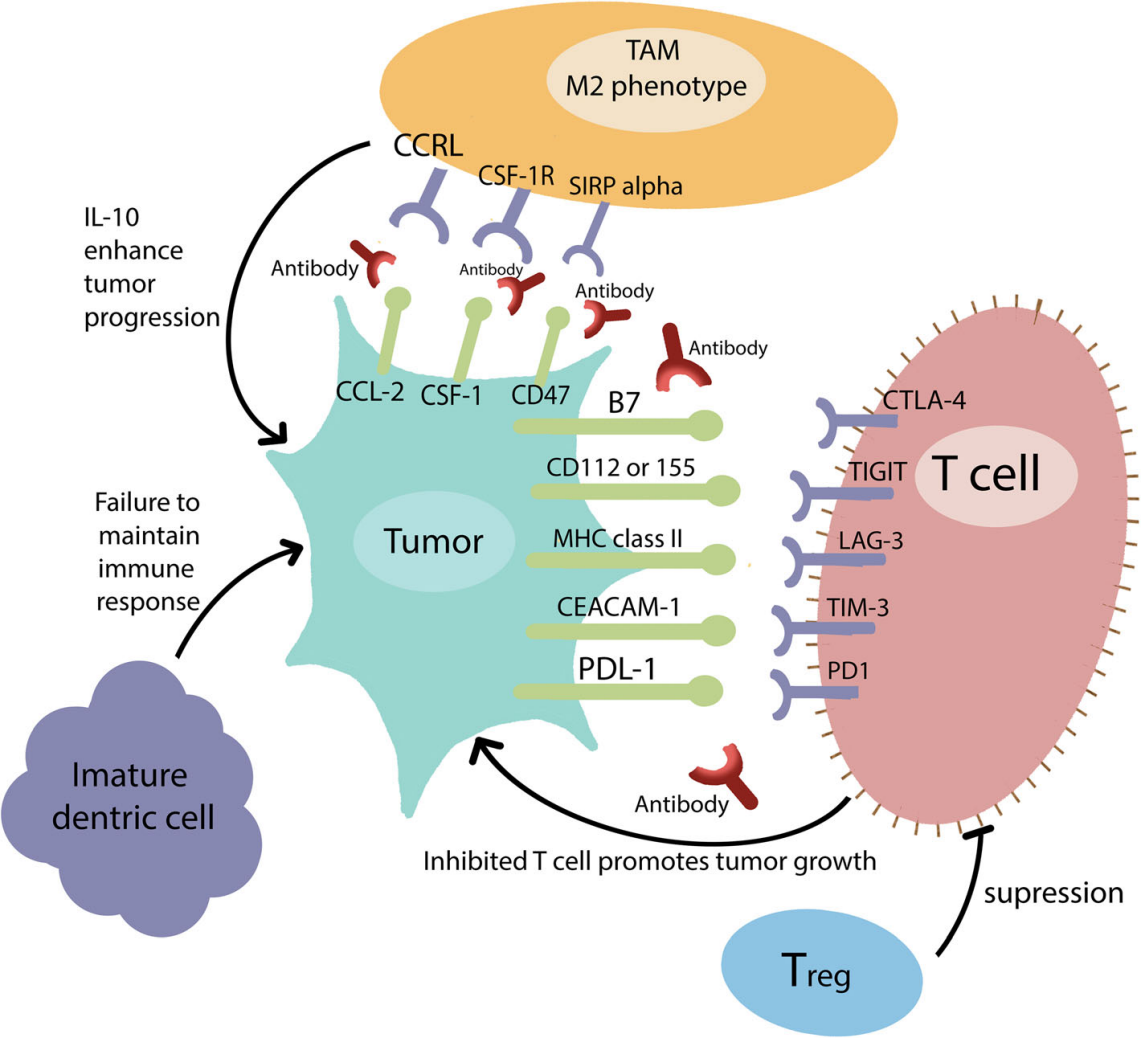
Tumor immune escape phase: The increased expression of checkpoint inhibitors on T cells surface and their corresponding ligands on tumor cells lead to remarkable inhibition of host immune response. Presence of regulatory T cells (Treg) and M2 phenotype TAM (tumor associated macrophages) in tumor microenvironment contribute to tumor progression through T cell suppression by the first and pro-tumor IL-10 secretion by the second

Several models have been developed to explain tumor immunosurveillance [59, 60]. These models divide the immune system’s response to cancer formation into 3 distinct phases: elimination, equilibrium, and escape. In the elimination phase, immune system can recognize and eliminate transformed cells. During the equilibrium phase, there is a generation of tumor cell variants with increased capacity of evading immunosurveillance due to the initial pressure by the immune system (Fig. 2). During the escape phase (Fig. 3), the emerging and evolved neoplastic cell variants are finally able to escape the immune system, leading to tumor growth and clinically evident disease.

To leverage the natural immune response and restore its elimination ability of thyroid tumor cells, crucial understanding of the tumor microenvironment and its complex interaction with the immune system is required. In Table 3, we highlight observations regarding specific patterns in tumor-associated immune cells within the thyroid cancer microenvironment, and how this might be used in anti-cancer future therapies.

**Table 3:** Patterns in tumor-associated immune cells within the thyroid cancer microenvironment

Immune Component (Cell type)	Studies in Thyroid Cancer (with references)
B cells	Antithyroid antibodies (secreted by B cells) are present in 18–40% of patients with PTC, 39% in those with benign thyroid nodules, and 10–14% of general population [138–142].
Mast Cells	PTC have been found to exhibit dense mast cell infiltration in comparison to normal thyroid tissues. This dense infiltration could be attributed to VEGF-A secretion by thyroid tumor cells which help in recruiting mast cells. Tumor recruiting mast cells play a role in tumor immune escape as these cells contribute to dedifferentiation, invasion, and angiogenesis of thyroid tumor through production of chemokines (CXCL1, CXCL10), histamine, and interleukin 8 [143–145].
T cells	Another proposed mechanism for tumor immune escape includes the overexpression of inhibitory checkpoint molecules in tumor associated T cells (Fig. 3). These molecules include PD-1, TIM-3, Lag-3, and TIGIT. The downstream inhibitory signaling of these molecules on T cells has been reviewed before [146] (Fig. 4). These molecules contribute to T cell dysfunction by affecting their production of the inflammatory cytokines IL-2, TNFα, and IFNγ. PD-1 molecule expression was associated with all classes of thyroid cancer with highest prevalence in anaplastic types. Regulatory T cells (Tregs) are a subpopulation of T cells known for their immune suppressive effects and tumor enhancing properties. Tregs exert these pro-tumor actions by expressing PD-1 and CTLA-4, another immune checkpoint modulator. Tregs were found in large amounts in advanced, locally-invasive DTC, lymph node metastases, and ATC. These findings support their role in tumor aggressiveness, and how targeting immune inhibitory Tregs can mediate better thyroid tumor control [74, 147–149].
Natural Killer cells (NK)	Patients with aggressive ATC or advanced and metastatic thyroid cancers were reported to have low peripheral blood NK cells in comparison to patients with benign lesions or other control patients. Introduction of IL-12 (an NK activating cytokine) in a murine model of BRAF-mutated thyroid cancer was helpful in restoring the tumor immune elimination properties. –Also, it is to be noted that NK cells could lyse anaplastic thyroid cells ex-vivo. It is hoped that these anti-tumoral activities of NK cells could be used in thyroid cancer immunotherapy [150–156].
Tumor associated macrophages (TAM)	These cells belong to the monocyte-macrophage lineage. There are two phenotypes of TAMs: M1 expressing IL-1, IL-12, and TNF-α, contributing to immune control over tumors; and M2 expressing IL-10 and CD163, promoting tumor progression and inhibition of tumor immune elimination. It was concluded that in PTC and poorly differentiated thyroid cancer, the density and presence of M2 TAMs correlated with tumor invasion and decreased survival. In anaplastic tumors, the TAMs form greater than 50% of tumor mass [157–159].
Dendritic cells	Immature dendritic cells expressing CD1a or S100 were found in PTC human tissue samples, and these cells failed to maintain an immune response to thyroid cancer cells [160–162]. A relation between TAMs and inability of dendritic cells to mature and present tumoral antigens has been proposed [163]. Such relation suggests that targeting inhibitory TAMs could enhance dendritic cells differentiation. However, this concept remains yet to be validated in more pre-clinical studies as well as clinical trials.

### Strategies for thyroid cancer immunotherapies

Many oncologic trials have focused on developing immunomodulating therapies to restore the functional ability of different immune cells against neoplastic cellsand, with promising results in several tumor types, including lung cancer, melanoma, and colon cancer [61, 62]. Recent efforts and advances in translational research led to proposing several strategies for immunomodulation in thyroid cancer and they will be reviewed here.

#### Inhibiting recruitment of tumor associated macrophages (TAM)

It was found that thyroid tumoral tissues expressed increased levels of *CSF-1* and *CCL-2* in human tissue samples [63]. These molecules are known to be chemoattractants to TAMs. Since TAM represent more than 50% of anaplastic thyroid cancer volume, and to a lesser extent that of advanced DTC, blocking and targeting CCL-2/CCR2 and *CSF-1/ CSF-1R* pathways represents a promising approach (Fig. 3). This approach proposes not only inhibiting the recruitment of pro-tumor M2 phenotype TAMs, but also their repolarization into the M1 antitumor phenotype [64–66]. Therefore, the concept of depleting and repolarizing TAM to enhance anti-tumor immune response is the subject of many ongoing trials. In NCT01346358, CSF-1R antibody LY3022855 (also known as IMC-CS4) is tested against advanced solid tumors. NCT01525602 is recruiting patients with advanced solid tumors to test the effect of CSF-1R inhibitor (PLX3397) plus paclitaxel. Both studies include a cohort of thyroid patients.

#### Identification of tumor specific antigen and tumor neoantigens

Creating a cancer vaccine was recently the purpose of the cancer “Moonshot 2020” project [67]. Identification of thyroid tumor specific or neoantigens could pave the path towards creating a successful vaccine for tumor specific dendritic cells and thyroid cancer specific T cells. Tumor-associated antigens (melanoma antigen encoding genes [MAGE], mucin-1 antigen [MUC1], and proto-oncogene c-MET) can be expressed along with thyroid specific proteins (thyroglobulin and thyroid peroxidase) in DTC [68–70].

Several studies showed that the burden of genetic alteration and thyroid tumor neo-antigen is more prevalent in poorly differentiated and anaplastic tumors than in early differentiated stages [71]. Dendritic cell (DC) vaccines targeting the carcinoembryonic antigen (CEA), which is commonly expressed in MTC, have shown some clinical promise in these patients [72]. In this study, mature DCs, generated from peripheral blood monocytes and loaded with calcitonin and carcinoembryonic antigen (CEA) were injected in a cohort of 7 patients. Calcitonin and CEA were remarkably decreased in 3/7 patients with one of these three showing complete regression of metastatic hepatic and pulmonary nodules [72].

Other efforts of clinical targeting specific thyroid antigens is also under investigation; NCT01856920 is testing GI-6207, a vaccine made from baker’s yeast targeting the CEA in patients with MTC. NCT02239861 is recruiting patients to test specific adoptive cytotoxic T cells targeting several tumor antigens (NY-ESO-1, MAGEA4, PRAME, survivin, and SSX) in patients with advanced solid tumors, including thyroid cancer patients.

#### Blocking and inhibiting immune checkpoints

**Fig. 4:**
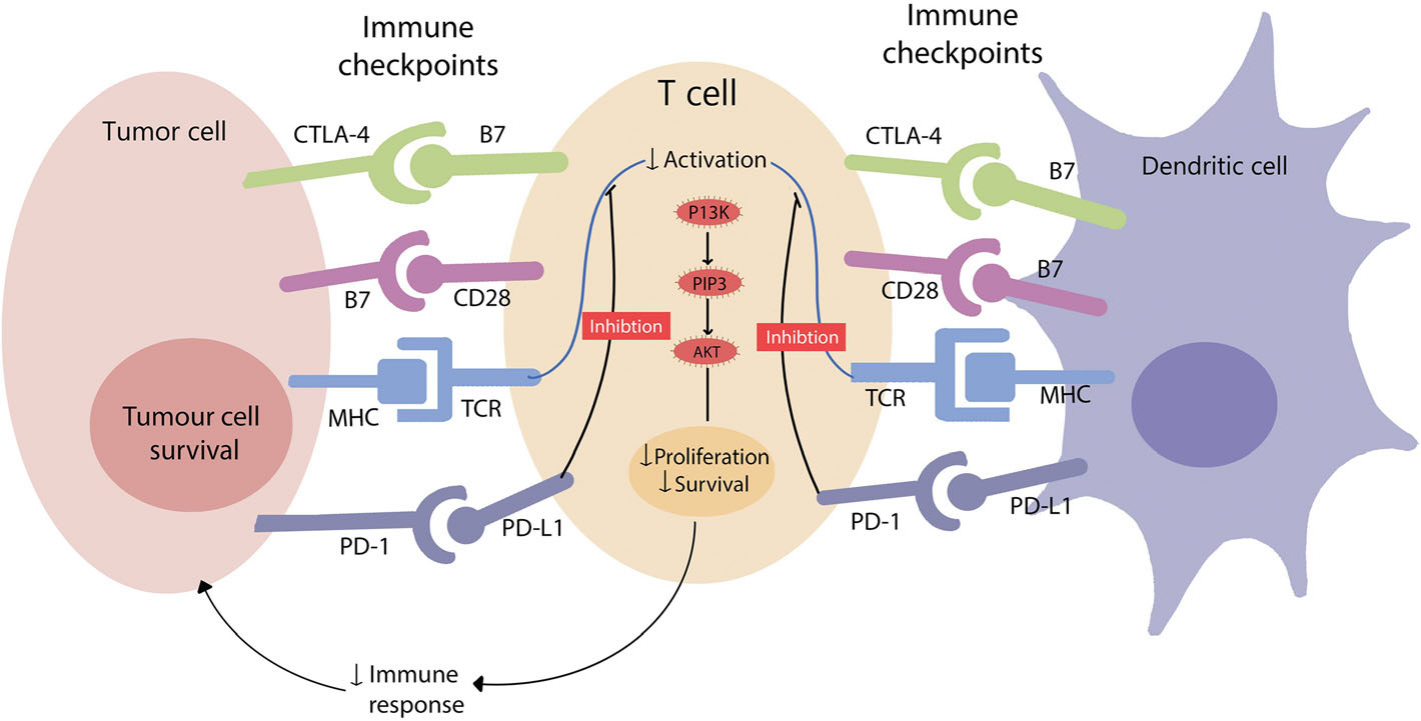
Immune checkpoints and their proposed mechanism of action in T cells: Several immune checkpoints are overexpressed on T cell surface in thyroid tumor microenvironment and their corresponding ligands on tumor cells or immature dendritic cells. Interaction of receptors and ligands lead to inhibition of the AKT pathway inside T cells and subsequently inhibition of T cell proliferation and division

The recent identification of blocking antibodies of CTLA-4 and PD-1 to their corresponding ligands (CD80/86 and PD-L1/PD-L2 respectively) represented a new hope in cancer immunotherapy [73]. Blocking these inhibitory pathways enhance the effector T cells and inhibit the regulatory suppressor cells (Fig. 4). Antibodies to CTLA-4 like Ipilimumab and Tremelimumab, and antibodies to PD-1 like Pembrolizumab and Novilumab, were approved by the FDA for treatment of several types of cancers including melanoma, non-small cell lung cancer, and renal carcinoma [73]. The increased frequency of PD-1(+) T cell in thyroid tumor-involved lymph nodes in PTC patients suggests potential utility for these checkpoint inhibitors in advanced thyroid cancers [74]. NCT02054806 is testing Pembrolizumab effect as monotherapy in advanced solid tumor patients including a cohort of thyroid cancer patients. Early results published in ASCO 2017 included a cohort of 22 thyroid patients in which 18 were radioactive iodine refractory; 7 received prior Sorafenib; and 1 received prior lenvatinib. Investigators reported 2/22 patients with PR, 12/22 patients with stable disease while 18/22 developed treatment related side effects most of them of grade 2–3. However, no patients discontinued or died from treatment adverse effects [75].

Dual targeting of the immune system in thyroid tumor microenvironment could, in theory, maximize the clinical benefits. This approach has been adopted by several trials: NCT02452424 is testing CSF-1R inhibitor (PLX3397) plus PD-1 inhibitor (Pembrolizumab) against advanced melanoma and other solid tumors, including thyroid. NCT02718911 is testing another CSF-1R inhibitor (LY3022855) plus Tremelimumab or Durvalumab (PD-1 inhibitors) in solid tumors.

#### Restoring the tumor phagocytic ability of TAM and enhancing tumor antigen presentation

Generally, most cancer cells, including thyroid cancers, were found to express an inhibitory receptor coined CD47, whose ligand is TAM signal-regulatory protein α (SIRPα). This receptor-ligand interaction leads to inhibiting the phagocytic ability of TAM, and impaired tumor antigen presentation by dendritic cells. In mouse models, blocking SIRPα/CD47 was successful in inducing tumor regression. Therefore, blocking CD47 by monoclonal antibodies or targeting SIRPα, represents an approach to reverse the immunoinhibitory effect of this pathway [76–78]. This concept was proven pre-clinically, where blocking CD47 in human thyroid cancer cell lines showed apoptotic effects [79]. However, clinical trials assessing CD47 targeting are needed to assess the clinical efficacy of this approach.

### MKIs and immunotherapy

Recent data showing a role for kinase inhibitors in immune modulation brings the promise that perhaps both strategies can be utilized simultaneously to achieve better responses in advanced thyroid cancer. Regulatory T cells express VEGFR-2, and both VEGF-A and *BRAF-V600E* are associated with upregulation of PD-1 expression, leading to inhibition of cytotoxic T cells [80–82].

Therefore, currently available MKIs could potentially reverse the inhibitory effects of pathologic kinase activities on the immune system.

For example, in an ex-vivo studies, Sorafenib reduced the number of immune inhibitory regulatory T cells (Treg) and hence, potentiating the immune response [83, 84]. Several clinical trials are currently assessing the effect of combining MKIs with immune therapy for refractory and advanced tumors. For example, Sulfatinib is an oral MKI targeting VEGFR, FGFR-1, and CSF1R, hence, potentially having a dual role in targeting angiogenesis and promoting immunomodulation. The first primary results of NCT02614495, an open label, two cohort, phase I and II trial assessing the role of Sulfatinib in advanced MTC and RAI-R DTC, were presented at ASCO 2017. In this trial, 18 patients (MTC: 6, DTC: 12) were assigned to Sulfatinib 300 mg once daily. There were a total of 4 confirmed partial responses, 3 in the DTC cohort and 1 in the MTC cohort; all other patients achieved stable disease. Treatment side effects were mostly grade 3–4 with no grade 5 toxicity observed. These side effects resulted in dose interruption in 11 patients and dose reduction in 5 patients. Combination therapy is being explored as well, for example NCT02501096 (phase IB/II trial) is currently recruiting patients with solid tumors (including thyroid cancer) to assess the maximum tolerated dose (MTD) for Lenvatinib in combination with Pembrolizumab during phase IB of the trial, and a subsequent expansion phase II trial will evaluate the safety and efficacy of this combination. NCT01988896 is assessing the effect of combining the PDL-1 inhibitor Atezolizumab, plus the mitogen-activated protein kinase (MAPK) inhibitor, Cobimetinib, in locally advanced or metastatic solid tumors. Other trials combining MKIs with immunotherapy that may redefine systemic therapy for advanced thyroid cancer are listed in Table 4.

**Table 4:** Immunotherapy trials addressing thyroid cancer

Immunological Target	Trial	Brief description
TAM	NCT01346358	CSF-1R antibody LY3022855 (also known as IMC-CS4) is tested in advanced solid tumors, including thyroid cancer
TAM	NCT01525602	Testing the effect of CSF-1R inhibitor (PLX3397) plus paclitaxel in patients with advanced solid tumors, including thyroid cancer
Dendritic cells	NCT01856920	Testing GI-6207, a vaccine made from baker’s yeast, targeting the CEA in patients with MTC
Dendritic cells	NCT02239861	Testing specific adoptive cytotoxic T cells targeting several tumor antigens (NY-ESO-1, MAGEA4, PRAME, survivin, and SSX) in patients with advanced solid tumors, including thyroid cancer patients
T cells	NCT02054806	Testing Pembrolizumab effect as monotherapy in advanced solid tumor patients, including a cohort of thyroid cancer patients
TAM	NCT02452424	Testing CSF-1R inhibitor (PLX3397) plus PD-1 inhibitor (Pembrolizumab) against advanced melanoma and other solid tumors, including thyroid
TAM	NCT02718911	Testing another CSF-1R inhibitor (LY3022855) plus Tremelimumab or Durvalumab (PD-1 inhibitors) in solid tumors
	NCT02614495	Open label - two cohorts - phase I, II trial assessing the role of Sulfatinib in advanced MTC and RAI-R DTC
T cells	NCT02501096	Phase IB/II trial, currently recruiting patients with solid tumors to assess the maximum tolerated dose (MTD) for Lenvatinib in combination with Pembrolizumab during phase IB of the trial. A subsequent expansion phase II trial will evaluate the safety and efficacy of this combination
T cells	NCT01988896	Testing the effect of combining the PDL-1 inhibitor Atezolizumab, plus the mitogen-activated protein kinase (MAPK) inhibitor, Cobimetinib, in Locally Advanced or Metastatic Solid Tumors
T cells	NCT01656642	Phase IB trial investigating PD-L1 antibody Atezolizumab plus mutant BRAF inhibitor Vemurafenib for patients with BRAFV600 mutation-positive metastatic melanoma (even though this trial has no thyroid patients, its results will help in designing future thyroid trials using such a combination based on pharmacodynamics and kinetics of this study)

## Future directions for scientific and clinical community

Based on current evidences and preliminary results of clinical trials assessing new treatment strategies, clustering patients with advanced thyroid cancers who failed conventional treatment options, according to their underlying molecular profile involved in disease development, will definitely help optimizing treatment decisions in clinical scenarios. This fact can be attributed to the widely varying molecular nature of different escape mechanisms from drugs targeting MAPK. The awaited clinical trials mentioned in this review (Additional file 1: Table S1) will definitely help implement new treatment strategies for these patients.

Also, there is clearly much work to be done to identify novel therapeutic targets and to develop strategies for treating advanced thyroid cancer. Pre-clinical data suggests a number of areas that could be developed in the coming years. For example, Histone Deacetylase Inhibitors (HDACIs) have been shown to increase expression of tumor suppressor genes in in vitro models of MTC [85–88]. Enthusiasm is tempered by two recent trials using the HDACI Valproic Acid in RAI-R DTC and ATC with negative results [89, 90], and yet other HDACIs may have potential based on very recent pre-clinical data,; for example CUDC-907, a dual inhibitor of HDACs and PI3k pathway, showing apoptotic effects in thyroid cancer cell lines in vitro and significant inhibition of growth and metastasis in a metastatic mouse model [91].

Another area to be developed is in the identification of biomarkers: a biomarker predicting the likelihood of clinical beneficence from targeted therapy is still lacking. One promising approach is looking at miRNAs, with certain expression patterns possibly providing a signature related to different clinicopathological features of thyroid cancer [92]. Finally, investigators are using kinome profiling, studying kinase complement of the human genome, combined with other transcriptional phosphoproteomics studies, to look for new treatment targets and strategies enhancing the currently available MKIs. One recent report identified SRC kinases as targets for invasive thyroid tumors using kinase phosphorylation assay [93]. Another report, using global phosphoproteomics analysis, identified CK2 (Caseine Kinsase 2) as a survival mechanism after BRAF-MEK signaling blockade [94]. These markers might be developed as tools to monitor response to different MKIs therapies targeting MAPK. An interesting report by Martinez et al. identified new molecular targets using mass spectrometry proteomics on a large scale [95]; in this study, TGFβ-induced protein ig-h3 (TGFBI) was found to be overexpressed in FTC. PTC samples, in comparison to FTC and normal tissue samples, was found to overexpress the extracellular protein Decorin, Tenascin, and AGR-2 [95]. These findings can provide future targets for new therapeutics.

## Conclusion

Thyroid cancer patients with advanced disease still do not have adequate treatment options, but the immediate and long-term future of therapy in this area looks bright. As research leads to a better understanding of underlying molecular changes in these patients, it should become increasingly possible to offer individualized, targeted therapy in their treatment protocols. With a number of MKIs being studied that can target the key pathways and escape mechanisms driving advanced thyroid cancer, and with the dawn of the age of immunotherapy, it can be hoped and perhaps even expected that soon novel therapeutics arise offering better long-term survival for these patients.

## Additional file

Additional file 1: **Table S1**. Ongoing clinical trials mentioned. (DOCX 16 kb)
