# Supplementary material for: Novel targeted therapies and immunotherapy for advanced thyroid cancers
Source: Mol Cancer. 2018 Feb 19;17:51. doi: 10.1186/s12943-018-0786-0 (PMC5817719; doi:10.1186/s12943-018-0786-0)
Supplement: Supplementary file 1 — Table S1. Ongoing clinical trials mentioned. (DOCX 16 kb) [file 12943_2018_786_MOESM1_ESM.docx]

Additional file 1: Table S1 Ongoing clinical trials mentioned

| Clinical Trial | Phase | Description | Primary endpoint |
| --- | --- | --- | --- |
| Targeted therapies trials | | | |
| NCT01947023 | 1 | Assessing Lapatinib (HER2/3 blocker) in combination with Dabrafenib (BRAF inhibitor) for patients with advanced DTC | To determine the maximum tolerated dose of lapatinib (lapatinib ditosylate) that can be used in combination of dabrafenib |
| NCT03065387 | 1 | pan-ERBB Inhibitor Neratinib is being assessed for its effect in advanced solid tumors harboring any HER mutation, including thyroid cancer | Maximum Tolerated Dose of Neratinib Combination Therapy |
| NCT02289144 | 2 | assess the role of Ceritinib in patients with advanced ATC with positive ALK abnormalities | Development of Progression |
| NCT01025453 | 2 | assessing the effect of Temsirolimus with Sorafenib in RAI-R thyroid cancer patients | Determine the objective response rate of the combination sorafenib and temsirolimus in I-131 refractory thyroid cancer |
| NCT01141309 | 2 | evaluating the combination of Everolimus (mTOR inhibitor) and Sorafenib | Determine the response rate of the combination sorafenib and everolimus |
| NCT01263951 | 2 | testing the effect of combination of Everolimus (mTOR inhibitor) and Sorafenib in DTC patients who progressed on Sorafenib therapy alone | To determine the efficacy of everolimus and sorafenib (progression free survival, objective response rate and stable disease) in patients with advanced differentiated thyroid cancer who have progressed on sorafenib alone |
| NCT02143726 | 2 | assesses the efficacy and safety of Sorafenib with or without Everolimus in treating patients with advanced, RAI-R Hurthle cell thyroid cancer | Progression Free Survival |
| NCT02289144 | 2 | assess the effect Ribociclib (targeting RB mutation) and Everolimus (mTOR inhibitor) in metastatic Rb+ ATC patients | Development of Progression |
| NCT01843062 | 3 | assess the efficacy of Selumetinib vs placebo in DTC patients | Complete remission rate in overall study population |
| NCT02034110 | 2 | assessing Efficacy of Dabrafenib (BRAF inhibitor) and Trametinib (MEK inhibitor) in 16 patients with BRAF V600E (ATC) | Overall response rate |
| NCT01723202 | 2 | assessing Efficacy of adding Trametinib to Dabrafenib as compared to Dabrafenib alone in BRAF mutated PTC patients | Overall objective response rate |
| NCT01843062 | 3 | placebo-controlled trial with Selumetinib in locally-advanced, recurrent, or metastatic thyroid cancer | Complete remission rate in overall study population |
| NCT02393690 | 2 | placebo-controlled trial with Selumetinib in RAI-avid recurrent and/or metastatic thyroid cancer | To determine the response rate at 6 months following treatment with 131I (iodine I-131) in combination with placebo or selumetinib for radioactive iodine-avid (RAIA) recurrent and/or metastatic thyroid cancer |
| NCT02152995 | 2 | placebo-controlled trial with MEK inhibitor Trametinib in locally-advanced, recurrent, or metastatic thyroid cancer | To evaluate the effect of trametinib on enhancing radioiodine (RAI) activity |
| NCT01346358 | 1 | CSF-1R antibody LY3022855 (also known as IMC-CS4) is tested in advanced solid tumors | Pharmacokinetics assessment |
| Immunotherapy trials | | | |
| NCT01525602 | 1 | test the effect of CSF-1R inhibitor (PLX3397) plus paclitaxel in patients with advanced solid tumors | Assess the safety of PLX3397 in combination with paclitaxel |
| NCT01856920 | 2 | testing GI-6207, a vaccine made from baker’s yeast targeting the CEA in patients with MTC | To test the safety and effectiveness of the GI-6207 vaccine for advanced medullary thyroid cancer |
| NCT02239861 | 1 | test specific adoptive cytotoxic T cells targeting several tumor antigens (NY-ESO-1, MAGEA4, PRAME, survivin, and SSX) in patients with advanced solid tumors, including thyroid cancer patients | evaluating the safety and feasibility of administering TAA-CTLs to patients with solid tumors |
| NCT02054806 | 1 | testing Pembrolizumab effect as monotherapy in advanced solid tumor patients including a cohort of thyroid cancer patients | assess the efficacy and safety of pembrolizumab (MK-3475) |
| NCT02452424 | 1/2a | testing CSF-1R inhibitor (PLX3397) plus PD-1 inhibitor (Pembrolizumab) against advanced melanoma and other solid tumors, including thyroid | Assess the safety of PLX3397 in combination with Pembrolizumab |
| NCT02718911 | 1 | testing CSF-1R inhibitor (LY3022855) plus Tremelimumab or Durvalumab (PD-1 inhibitors) in solid tumors | Assess the maximum tolerated dose |
| NCT02614495 | 1/2 | assessing the role of Sulfatinib in advanced MTC and RAI-R DTC | assess the efficacy and safety of Sulfatinib 300 mg Sulfatinib in advanced Medullary Thyroid Carcinoma ( MTC) and iodine-refractory differentiated thyroid carcinoma (DTC) |
| NCT02501096 | 1b/2 | assess the maximum tolerated dose (MTD) for Lenvatinib in combination with Pembrolizumab during phase IB of the trial, and a subsequent expansion phase II trial will evaluate the safety and efficacy of this combination | Assess maximum tolerated dose, objective response rate, and dose limiting toxicity |
| NCT01988896 | 1 | assessing the effect of combining the PDL-1 inhibitor Atezolizumab, plus the mitogen-activated protein kinase (MAPK) inhibitor, Cobimetinib, in locally advanced or metastatic solid tumors | Assess dose-limiting toxicities and maximum tolerated dose |
| NCT01346358 | 1 | CSF-1R antibody LY3022855 (also known as IMC-CS4) is tested in advanced solid tumors, including thyroid cancer | establish the safety profile and characterize the pharmacokinetic profile of IMC-CS4 |
| NCT01525602 | 1 | Testing the effect of CSF-1R inhibitor (PLX3397) plus paclitaxel in patients with advanced solid tumors, including thyroid cancer | Assess incidence of adverse events, response to treatment, and progression-free survival |
| NCT01856920 | 2 | Testing GI-6207, a vaccine made from baker’s yeast, targeting the CEA in patients with MTC | test the safety and effectiveness of the GI-6207 vaccine for advanced medullary thyroid cancer |
| NCT02239861 | 1 | Testing specific adoptive cytotoxic T cells targeting several tumor antigens (NY-ESO-1, MAGEA4, PRAME, survivin, and SSX) in patients with advanced solid tumors, including thyroid cancer patients | Assess dose-limiting toxicity |
| NCT02054806 | 1 | Testing Pembrolizumab effect as monotherapy in advanced solid tumor patients, including a cohort of thyroid cancer patients | Assess overall response rate, progression-free survival, and overall survival |
| NCT02452424 | 1/2 | Testing CSF-1R inhibitor (PLX3397) plus PD-1 inhibitor (Pembrolizumab) against advanced melanoma and other solid tumors, including thyroid | Assess safety and objective response rate |
| NCT02718911 | 1 | Testing CSF-1R inhibitor (LY3022855) plus Tremelimumab or Durvalumab (PD-1 inhibitors) in solid tumors | Assess maximum tolerated dose and response rates |
| NCT02614495 | 2 | assessing the role of Sulfatinib in advanced MTC and RAI-R DTC | Assess objective response rate |
| NCT02501096 | 1b/2 | currently recruiting patients with solid tumors to assess the maximum tolerated dose (MTD) for Lenvatinib in combination with Pembrolizumab during phase IB of the trial. A subsequent expansion phase II trial will evaluate the safety and efficacy of this combination | Assess maximum tolerated dose, objective response rate, and dose-limiting toxicity |
| NCT01988896 | 1 | Testing the effect of combining the PDL-1 inhibitor Atezolizumab, plus the mitogen-activated protein kinase (MAPK) inhibitor, Cobimetinib, in Locally Advanced or Metastatic Solid Tumors | Assess maximum tolerated dose and dose-limiting toxicity |
| NCT01656642 | 1b | investigating PD-L1 antibody Atezolizumab plus mutant BRAF inhibitor Vemurafenib for patients with BRAFV600 mutation-positive metastatic melanoma (even though this trial has no thyroid patients, its results will help in designing future thyroid trials using such a combination based on pharmacodynamics and kinetics of this study) | Assess dose limiting toxicity and adverse events |
